# Why Is the Invasive Plant *Sphagneticola trilobata* More Resistant to High Temperature Than Its Native Congener?

**DOI:** 10.3390/ijms22020748

**Published:** 2021-01-13

**Authors:** Minling Cai, Xiaohua Lin, Jindi Peng, Junjie Zhang, Minghao Chen, Jundong Huang, Lihua Chen, Feng Sun, Wenqiao Ding, Changlian Peng

**Affiliations:** Guangzhou Key Laboratory of Subtropical Biodiversity and Biomonitoring, Guangdong Provincial Key Laboratory of Biotechnology for Plant Development, College of Life Sciences, South China Normal University, Guangzhou 510631, China; 2020010178@m.scnu.edu.cn (M.C.); 20172521046@m.scnu.edu.cn (X.L.); 20172521085@m.scnu.edu.cn (J.P.); 2019022505@m.scnu.edu.cn (J.Z.); 2017022212@m.scnu.edu.cn (M.C.); 2019022493@m.scnu.edu.cn (J.H.); 2020022828@m.scnu.edu.cn (L.C.); 20198889@m.scnu.edu.cn (F.S.); 2018022497@m.scnu.edu.cn (W.D.)

**Keywords:** high temperature stress, Sphagneticola, gene expression, photosynthesis

## Abstract

Climate change and invasive alien species threaten biodiversity. High temperature is a worrying ecological factor. Most responses of invasive plants aimed at coping with adversity are focused on the physiological level. To explore the molecular mechanisms underlying the response of an invasive plant (*Sphagneticola trilobata* L.) to high temperature, using a native species (*Sphagneticola calendulacea* L.) as the control, relevant indicators, including photosynthetic pigments, gas exchange, chlorophyll fluorescence, the antioxidant system, and related enzyme-coding genes were measured. The results showed that the leaves of *S. calendulacea* turned yellow, photosynthetic pigment content (Chl *a*, Chl *b*, Car, Chl) decreased, gas exchange (P_n_) and chlorophyll fluorescence parameters (F_v_/F_m_, Φ_PSII_) decreased under high temperature. It was also found that high temperature caused photoinhibition and a large amount of ROS accumulated, resulting in an increase in MDA and relative conductivity. Antioxidant enzymes (including SOD, POD, CAT, and APX) and antioxidants (including flavonoids, total phenols, and carotenoids) were decreased. The qPCR results further showed that the expression of the *PsbP*, *PsbA,* and *RubiscoL*, *SOD*, *POD*, *CAT*, and *APX* genes was downregulated, which was consistent with the results of physiological data. Otherwise, the resistance of *S. trilobata* to high temperature was better than that of *S. calendulacea*, which made it a superior plant in the invasion area. These results further indicated that the gradual warming of global temperature will greatly accelerate the invasion area of *S. trilobata*.

## 1. Introduction

Climate change poses potential major threats to global biodiversity and ecosystem functions. The global average temperature is expected to increase by 1.4–4.3 °C by the end of the twenty-first century [1]. Global warming and rising temperatures will cause more frequent and extremely high temperatures in many parts of the world. High temperature (HT) is a worrying ecological factor. When a plant is exposed abruptly to temperatures above the normal optimum, homeostasis in the cell is disturbed, some catalytic enzymes, gradually lose their activity, and the cell structure is damaged, resulting in plant growth reduction, changes in photosynthetic efficiency, oxidative stress, and even death under extreme conditions [2,3,4]. Therefore, high-temperature stress is among the main abiotic stresses limiting plant productivity.

Alien species invasion is recognized as an important part of global environmental change. Throughout the 21st century, climate change and invasive alien species have been threatening biodiversity [5,6]. In many cases, climate warming may facilitate the spread of invasive plants by increasing their growth and competitive ability [7]. *Sphagneticola trilobata* (L.) Pruski (Asteraceae), listed by the IUCN among the top 100 of most alien invasive species in the world, is a perennial creeping herb and native to South America [8]. It is now widely distributed in many countries and regions of Southeast Asia and the Pacific. In the 1970s, it was introduced into China as a ground cover plant and soon escaped into the wild. At present, it is widely distributed in Southeast China, coastal areas, islands, and other areas [9]. In South China, *S. trilobata* has been expanding continuously, showing strong tolerance ability under high temperature in summer, crowding out *Sphagneticola calendulacea* (L.) Pruski and forming a single dominant species, which seriously threatens the biodiversity of its indigenous congener [10]. This is closely related to its strong resistance mechanism.

Photosynthesis is the most sensitive biological process affected by high-temperature stress, and is closely related to plant metabolism and growth performance. The photosystem II (PSII) reaction center and CO_2_ assimilation are the main targets that are vulnerable to high temperature stress. The PSII reaction center is one of the most heat-sensitive components of the photosynthetic apparatus, and the expression of genes involved in regulation of photosynthesis will be affected by high temperature [11]. It has been found that the electron donor side of PSII is a primary damage site, and oxygen-evolving complex (OEC) activities were found to decrease under high temperature [4]. High temperature can also affect the process of carbon assimilation, by inhibiting the activities of ribulose-1,5-bisphosphate carboxylase/oxygenase (rubisco) and rubisco activase (RA) and reducing the photosynthetic rate of plants [12]. Apart from this, high temperature increases the generation of reactive oxygen species (ROS), including singlet oxygen (_1_O^2^), superoxide anion (O_2_^−^), hydrogen peroxide (H_2_O_2_), and hydroxyl radical (•OH). Various subcellular organelles including chloroplasts, mitochondria, and peroxisomes are the common sites of ROS production. The excessive production of ROS can damage enzymes, cellular proteins, DNA and RNA, eventually resulting in the death of cells [13]. Membranes are the targets of HT stress and membrane lipid composition is a crucial factor for temperature tolerance or susceptibility. HT stress causes damage to thylakoid membranes, increase lipid peroxidation, and cause membrane damage [14]. Plants have developed an efficient antioxidative system with enzymatic and non-enzymatic antioxidants, which can reduce ROS up to a certain level. These antioxidant enzymes are superoxide dismutase (SOD), catalase (CAT), peroxidase (POD), ascorbate peroxidase (APX), monodehydroascorbate reductase (MDHAR), glutathione reductase (GR), and dehydroascorbate reductase (DHAR), which are found in different organelles or subcellular compartments. Previous studies have shown that the changes in the gene expression of antioxidant enzymes may contribute to plant molecular adaptation, and maintaining a high level of antioxidant transcripts may play a key role in protecting plants from oxidative stress [15]. Zhao et al. found that the heat-acclimated plant *Orchardgrass* maintained high levels of gene expression encoding SOD, CAT, and POD enzymes under heat stress [16]. In addition, Flavonoids, carotenoids, ascorbate, tocopherols, phenolics, and glutathione serve as potent non-enzymatic antioxidants in plants. A previous study showed that the thermal stability of the photosynthetic apparatus of *S. trilobata* is higher than that of the *S. calendulacea.* On exposure to HT, *S. trilobata* has an effective regulatory mechanism in the energy partitioning of PSII complexes by increasing the xanthophyll-dependent thermal dissipation (Φ_NPQ_) and decreasing slightly the proportion of constitutive thermal dissipation (Φ_f,D_) [10]. However, the mechanism of antioxidant activity has not been reported in depth. Many researchers have focused on the physiological level of two Sphagneticola species under adverse conditions [17,18]. Nevertheless, the expression patterns of genes involved in photosynthesis and antioxidant systems in response to adversity is not clear. Thus, we compared the antioxidant activity, photosynthetic and gene expression patterns of two Sphagneticola species under high temperature. The insights from this study will help us further study the molecular mechanism of thermoregulation of *S. trilobata*.

## 2. Results

### 2.1. Changes in Plant Appearance and Photosynthetic Pigments under HT Treatment

**Figure 1:**
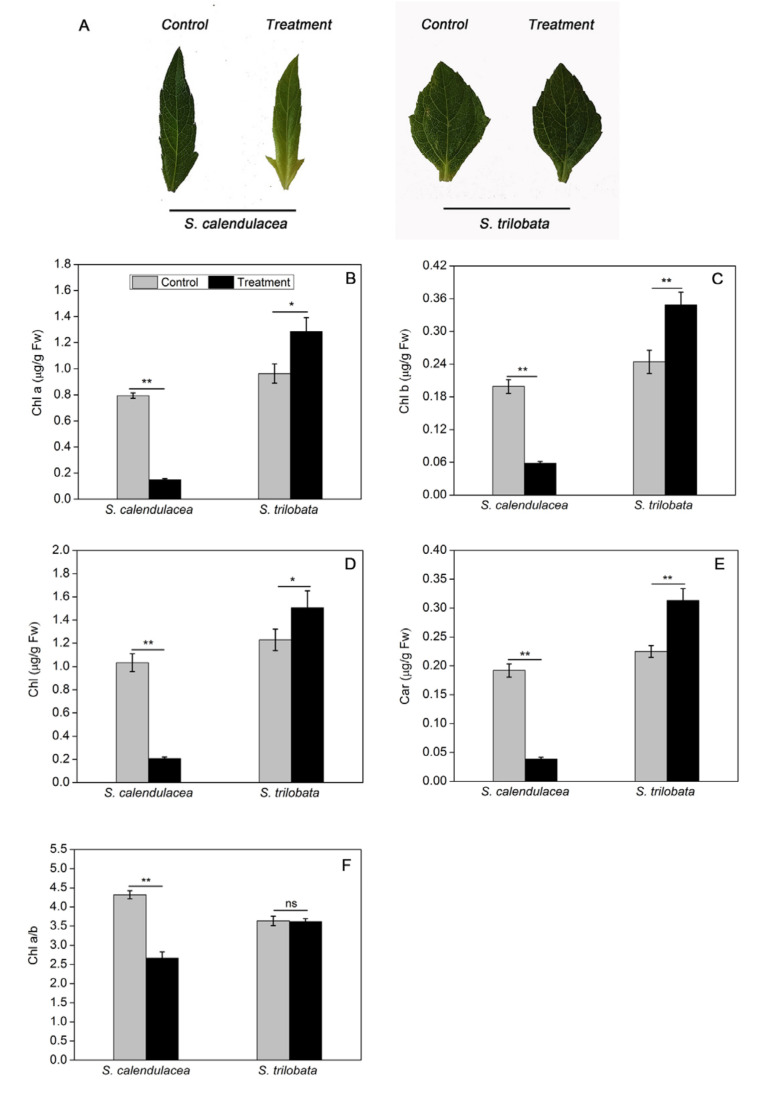
Effect on appearance (**A**), Chl *a* (**B**), Chl *b* (**C**), Chl (**D**), Car (**E**) content and Chl a/*b* (**F**) in two Sphagneticola species under HT treatment (35 ± 1 °C). The data represent the mean ± SE (*n* = 5). Asterisks above columns indicate statistical significance for comparisons between HT and room temperature. (** *p* < 0.01 and * *p* < 0.05).

After 22 days of treatment, we observed that the leaves of *S. trilobata* exposed to HT were greener than those of the control group (25 ± 1 °C). In contrast, *S. calendulacea* leaves exhibited an obvious yellow color under HT treatment, especially in the leaf base region (Figure 1A). Compared with the control group, different trends were observed in the variations in photosynthetic pigments in the two Sphagneticola species under HT treatment. The chlorophyll *a* (Chl *a*), chlorophyll *b* (Chl *b*), total chlorophyll (Chl), carotenoids (Car), and chlorophyll *a/b* (Chl *a/b*) in the leaves of *S. trilobata* exposed to HT were all increased, while they decreased significantly in the leaves of *S. calendulacea* (*p* < 0.01), coinciding with the leaf appearance (Figure 1B–F).

### 2.2. Changes in Photosynthetic Capacity under HT Treatment

**Figure 2:**
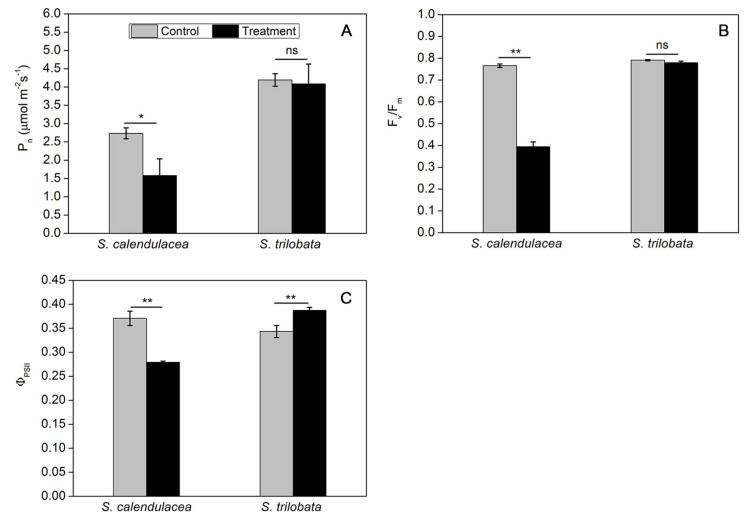
Effect on P_n_ (**A**), F_v_/F_m_ (**B**), and Φ_PSII_ (**C**) in two Sphagneticola species under HT treatment (35 ± 1 °C). The data represent the mean ± SE (*n* = 5). Asterisks above columns indicate statistical significance for comparisons between HT and room temperature. (** *p* < 0.01 and * *p* < 0.05).

Under HT treatment, we found that there was a significant decrease reduction of 42.2% in net photosynthetic rate (P_n_) values of *S. calendulacea* (*p* < 0.05). However, no significant differences were observed in the P_n_ values of *S. trilobata* (Figure 2A). The photosynthetic efficiency of plants is closely related to the electron transfer of chloroplasts. With respect to the control treatment, we found that *S. calendulacea* and *S. trilobata* showed decreases in maximum photochemical efficiency of PSII (F_v_/F_m_) of 48.5% and 1.4% under HT treatment, respectively (Figure 2B). We also observed that the operational photochemical efficiency (Φ_PSII_) increased in *S. trilobata* but was significantly inhibited in *S. calendulaceas* with a decrease range of 24.6% during the exposure to high temperature (Figure 2C).

### 2.3. Changes in Reactive Oxygen Species (ROS) and Cellular Membrane Stability under HT Treatment

**Figure 3:**
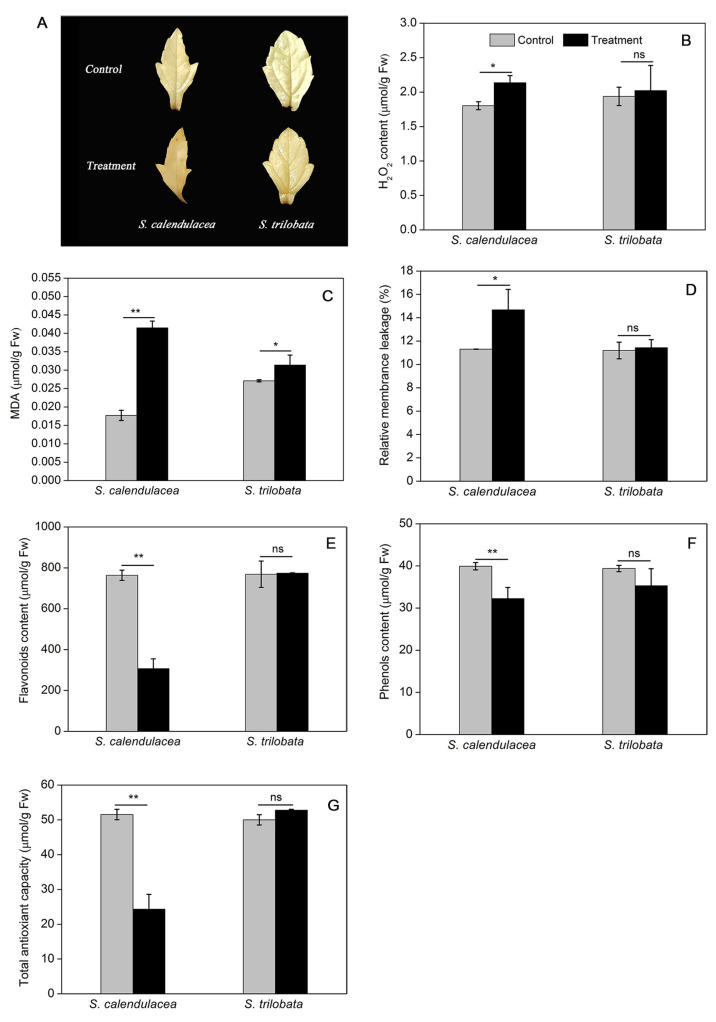
Results of H_2_O_2_ histochemical staining (**A**), H_2_O_2_ content (**B**), MDA content (**C**), membrane leakage rate (**D**) and non-enzymatic antioxidants, including flavonoids (**E**), total phenols (**F**) and TAC (**G**), in two Sphagneticola species under HT treatment (35 ± 1 °C). The data represent the mean ± SE (*n* = 5). Asterisks above columns indicate statistical significance for comparisons between HT and room temperature (** *p* < 0.01 and * *p* < 0.05).

Generally, ROS are produced in cell under a variety of environmental stress conditions, which can lead to oxidative stress in plants. The histochemical staining results showed that H_2_O_2_ accumulated in the leaves of the *S. calendulacea* was more than that of *S. trilobata* under HT treatment, and was mainly distributed at the leaf base region (Figure 3A). The H_2_O_2_ content in two Sphagneticola species was further quantified using a colorimetric assay. We found that H_2_O_2_ content was also significantly increased in *S. calendulacea* (*p* < 0.05) while slightly increased in *S. trilobata* (Figure 3B). The malondialdehyde (MDA) content of the two Sphagneticola species increased upon exposure to high temperature, and the change ranges of *S. trilobata* (15.9%) was much smaller than that of *S. calendulacea* (134.5%) (Figure 3C). It was also found that the membrane leakage rate increased in *S. calendulacea* by 29.8%, whereas there was no significant difference in the leaves of *S. trilobata* (Figure 3D).

### 2.4. Changes in Non-Enzymatic Antioxidant Content and Antioxidant Enzyme Activity under HT Treatment

Flavonoids and total phenols are antioxidants that exist in plants to scavenge ROS, and the total antioxidant capacity (TAC) of leaves is the comprehensive performance of the main antioxidant substances in the leaves. The results showed that, compared with the control treatment, no significant differences in the values of flavonoids, total phenols or TAC were observed for *S. trilobata* under HT treatment. However, *S. calendulacea* showed decreased flavonoids, total phenols, and TAC values of 59.8%, 19.2%, and 52.8%, respectively (Figure 3E–G).

**Figure 4:**
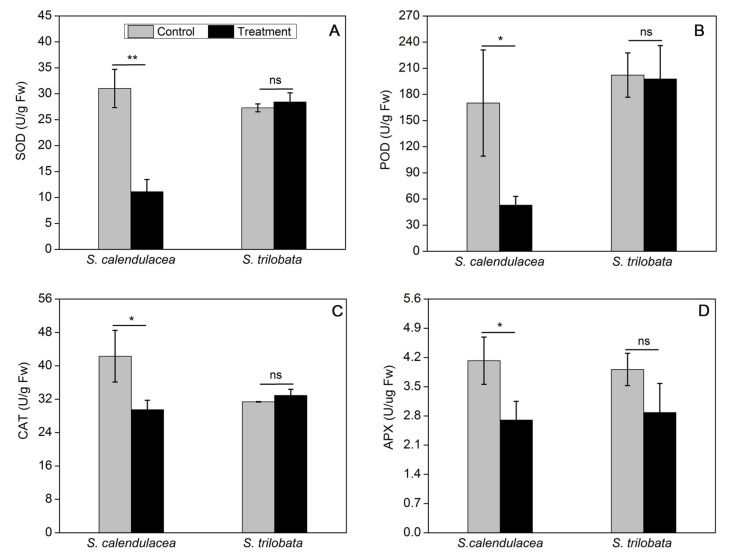
Effect on antioxidant enzyme activities including SOD (**A**), POD (**B**), CAT (**C**), and APX (**D**) in two Sphagneticola species under HT treatment (35 ± 1 °C). The data represent the mean ± SE (*n* = 5). Asterisks above columns indicate statistical significance for comparisons between HT and room temperature. (** *p* < 0.01 and * *p* < 0.05).

Moreover, we observed that SOD, POD, and CAT activities of *S. trilobata* did not change under HT treatment, but all of them were greatly reduced in *S. calendulacea* with change ranges of 64.2%, 68.9%, and 30.3%, respectively (Figure 4A–C). Unlike SOD, POD, and CAT, the decreased activity of APX was obtained in two Sphagneticola species under HT treatment, whereas the decreased range of *S. trilobata* (26.3%) was much smaller than that of *S. calendulacea* (34.5%) (Figure 4D). These results are in agreement with the previous reports regarding the changes in non-enzymatic antioxidants content under HT treatment.

### 2.5. Changes in the Expression of Genes Encoding Photosynthesis and Antioxidant Enzymes under HT Treatment

**Figure 5:**
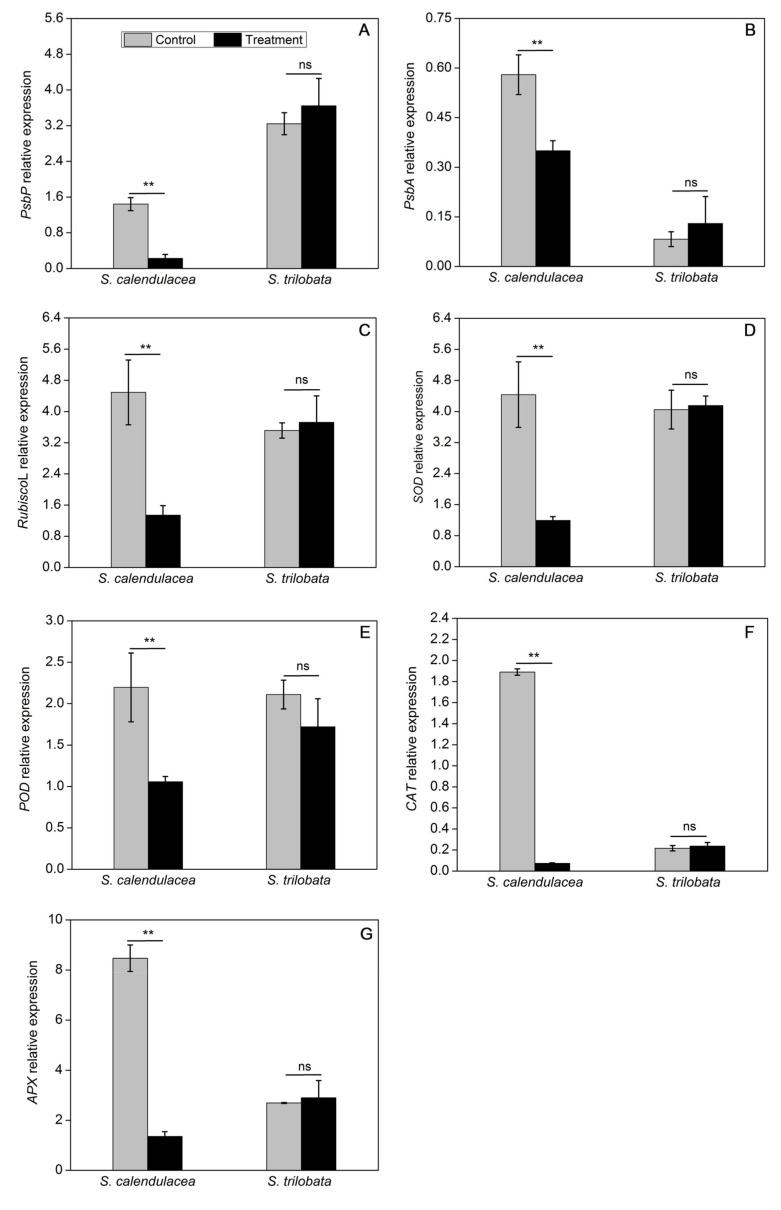
Effect on relative expression levels of photosynthesis-related genes, including *PsbP* (**A**), *PsbA* (**B**), *RubiscoL* (**C**), and antioxidant enzyme-related genes, including *SOD* (**D**), *POD* (**E**), *CAT* (**F**), and *APX* (**G**) in two Sphagneticola species under HT treatment (35 ± 1 °C). The data represent the mean ± SE (*n* = 5). Asterisks above columns indicate statistical significance for comparisons between HT and room temperature. (** *p* < 0.01).

The expression of genes encoding photosynthesis and antioxidant enzymes was measured by the method of qRT-PCR. The results showed that the expression levels of photosynthesis-related genes, including *PsbP*, *PsbA*, and *Rubisco L*, were slightly increased in *S. trilobata* under HT treatment, but there were no significant differences in all of them. In contrast, the transcript levels of photosynthesis-related genes decreased by 1.6–7-fold in *S. calendulacea* under HT treatment (Figure 5A–C). The expression pattern of antioxidant enzyme-related genes was similar to that of photosynthesis-related genes. Therefore, SOD, POD, CAT, and APX gene expression levels were also not significantly different in *S. trilobata*, while these levels were all decreased in *S. calendulacea* and the expression of CAT was most obviously decreased by approximately 27.0-fold under HT treatment compared to the control treatment (Figure 5D–G). The expression of these key enzyme genes at the transcription level was consistent with a previous report related to photosynthetic capacity (Figure 2A) and antioxidant enzyme activity (Figure 4).

## 3. Discussion

Photosynthesis is considered to be the most important photochemical reaction in plants and is highly sensitive to temperature. In this study, the expression of genes in the photosynthesis pathway of *S. trilobata* were not significantly different under HT stress. Conversely, *PsbA* and *PsbP* gene expression in the leaves of *S. calendulacea* was significantly decreased under HT stress (Figure 5A,B). A low level of *PsbA* gene expression has been shown to negatively influence the D1 content in PSII [19] and decrease the photosynthesis capability [20]. The OEC protein is encoded by the *PsbP* gene, and its damage will also inevitably affect the heat tolerance of PSII under HT stress [21]. HT stress further inhibited the electron transfer from the OEC protein to the PSII acceptor side [22]. The result of this study showed that F_v_/F_m_ and Φ_PSII_ in *S. trilobata* were close to or even slightly higher than normal temperature, while all of them were significantly decreases in *S. calendulacea* (Figure 2B,C). It was suggested that compared with *S. trilobata,* the leaves of *S. calendulacea* were the most seriously damaged under HT stress. Similar results were also found in tomato seedlings [23]. Studies have shown that the capacity for ribulose-1,5-diphosphate (RuBP) regeneration could be dramatically inhibited by the disruption of electron transfer and the inactivation of the OEC enzyme [24,25]. The RT-qPCR results demonstrated that the *RubiscoL* gene expression was significantly decreased in *S. calendulacea* under HT stress (Figure 5C), thus limiting the rate of CO_2_ fixation [12]. This is in accordance with the results of P_n_ (Figure 2A). Liu et al. have found that lower quantities of the photosynthetic pigments may inhibit the photosynthetic capacity [26]. The results showed that HT stress reduced Chl contents in the leaves of *S. calendulacea* (Figure 1B–F) and limited the absorption of light energy [27]. Particularly, there was a significant increase in the photosynthetic pigment content in *S. trilobata* and its leaves remained green, indicating the photosynthetic capacity of *S. trilobata* was basically not affected by HT stress. These results are consistent with Song et al. [10].

It has been seen that HT increases the generation of ROS [28]. The results of this study showed that there was no accumulation of ROS in *S. trilobata* at HT, while the situation in *S. calendulacea* was contrary (Figure 3A,B). Some studies have shown that ROS accumulate in plants under stress and attack unsaturated fatty acids in cells, aggravate membrane peroxidation, and lead to membrane system damage and cell damage [29]. The MDA content and relative conductivity of *S. calendulacea* both increased significantly under HT stress, while MDA content in *S. trilobata* increased slightly, and its relative conductivity was not significantly increased compared with the normal temperature (Figure 3C,D). These results suggested that compared with *S. calendulacea*, *S. trilobata* was less injured, and the cell membrane stability was stronger under HT stress. Maintenance of membrane integrity is a major mechanism by which plants cope with HT stress [30]. Furthermore, some studies have also shown that excessive accumulation of ROS can bleach photosynthetic pigments, affecting the absorption of light energy [31,32], which could explain the significant reduction in chlorophyll content of *S. calendulacea* leaves and the phenomenon of yellowing under HT stress (Figure 1A).

To protect plants from oxidative stress and maintain normal cell function, antioxidant enzymes including SOD, POD, CAT, and APX, play the role of ROS scavengers in plants. We can further understand the molecular adaptation of plants to HT stress by studying the gene expression underlying the change in antioxidant enzyme activity. The results indicated that the SOD, POD, CAT, and APX genes of *S. calendulacea* showed low expression at the transcription level (Figure 5D–G), and the antioxidant enzyme activity was also decreased under HT stress (Figure 4A–D). Studies have demonstrated that various environmental stresses lead to H_2_O_2_ production, and its excessive production causes the regulation of POD, CAT, and APX genes expression [33]. It has also been found that a decrease in CAT and APX activity will greatly weaken the protective effect of the antioxidant system, resulting in an increase in the active oxygen concentration [34]. The decrease in CAT and POD enzyme activities could not timely convert H_2_O_2_ into H_2_O and O_2_ that was harmless to cells. In contrast, under HT treatment, the antioxidant enzyme gene expression and its regulated enzyme activity of *S. trilobata* remained at a high levels as observed under normal temperature, which was an important reason for its stronger ROS-scavenging ability. In addition, non-enzymatic antioxidants including flavonoids (Figure 3E), total phenols content (Figure 3F), and TAC (Figure 3G) decreased significantly, indicating that scavenging capacity of ROS was much lower than that of *S. trilobata*. Moreover, carotenoid content increased in *S. trilobata* but decreased significantly in *S. calendulacea* under HT stress (Figure 1E). Studies have found that carotenoids could act as endogenous antioxidants of cells, absorbing residual energy, quenching active oxygen, and preventing membrane peroxidation [35]. Therefore, the destruction of the antioxidant system, which failed to remove ROS in a timely manner, was the main reason that the degree of oxidative stress in *S. calendulacea* is more serious than that of in *S. trilobata.*

**Figure 6:**
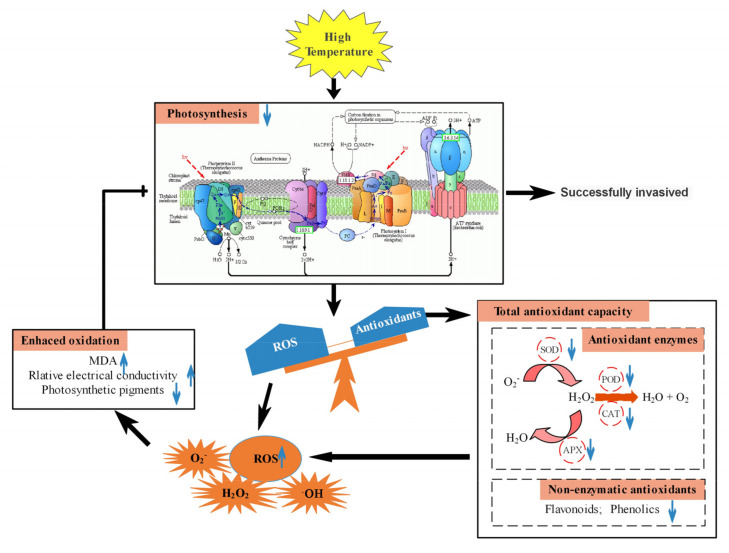
Photosynthetic response of two Sphagneticola species under HT stress. HT inhibited the expression of key PSII genes including *PsbP*, *PsbA,* and *RubiscoL*, decreased F_v_/F_m_ and Φ_PSII_, and greatly reduced the photosynthetic capacity of *S. calendulacea*. High temperature results in light inhibition, which leads to the accumulation of ROS. However, the scavenging ability of the enzymatic and non-enzymatic antioxidant systems of *S. calendulacea* was decreased and ROS could not be removed in time. On the one hand, the decreased antioxidant enzyme activity was due to the down-regulation of *SOD*, *POD*, *CAT,* and *APX* genes expression. On the other hand, the antioxidants contents including flavonoids, total phenols, and carotenoids were also decreased. This accumulation of ROS further caused an increase in MDA and relative conductivity, thus increasing the degree of membrane peroxidation and pigment bleaching in photosynthetic organs. However, the resistance of *S. trilobata* to cope with HT stress was better than that of *S. calendulacea*.

In summary, as a heat-sensitive plant, the ability of *S. calendulacea* to resist HT stress in South China was far lower than that of *S. trilobata*. The main reason was the difference of photosynthesis in response to HT stress (Figure 6). The resistance of *S. trilobata* to cope with HT stress was better than that of *S. calendulacea*, which made it a single superior population in the invasion area. These results further indicated that the gradual warming of global temperature will greatly accelerate the invasion area of *S. trilobata.*

## 4. Materials and Methods

### 4.1. Plant Materials, Cultivation, and HT Treatment

*Sphagneticola trilobata* and *Sphagneticola calendulacea* were collected from the campus of South China Normal University (Guangzhou, China). The experimental plant materials were propagated from segments that had two stem nodes. After rooting and growing 2–3 young leaves, the segments were transplanted into pots. One plant of each kind per pot was cultured using hydroponics in a greenhouse under natural light conditions, with a temperature of 25 ± 1 °C, 50–70% relative humidity, and a 12 h photoperiod. After one month of growth, uniform seedlings with the same size and height were selected for study. One half of the materials were randomly selected for HT treatment (35 ± 1 °C). Materials grown at room temperature (25 ± 1 °C) were used as the control group. Control and treatment groups were set for each species of Sphagneticola. After 22 days of HT treatment, the phenotype was recorded and the relevant physiological indexes were measured.

### 4.2. Gas Exchange and Chlorophyll Fluorescence Measurements

Gas exchange was measured using the Li-6400 portable photosynthesis system (LI-COR, Inc., Lincoln, NE, USA). Before the measurement, the leaves were acclimated for 5–10 min at a photosynthetic active radiation (PAR) of 800 μmol m^−2^s^−1^ and a CO_2_ concentration was maintained at 400 µmol mol^−1^ in this study. Once the steady-state gas exchange rates were observed at these conditions, the instrument automatically calculates and records the net photosynthetic rate (P_n_).

The chlorophyll fluorescence parameters were tested by a chlorophyll fluorescence imaging system (CF imager, Technical Ltd., Colchester, UK). Before each test of the minimum fluorescence (F_o_) and maximum fluorescence (F_m_), the leaves were dark-adapted for 20 min. The maximum quantum yield (F_v_/F_m_) of PSII photochemistry was calculated using the following formula: (F_m_—F_o_)/F_m_. The steady-state fluorescence (F_s_) and the maximum fluorescence (F_m_’) in the light-adapted state were measured at 800 μmol m^−2^s^−1^ PPFD. The actual photochemical efficiency (Φ_PSII_) was calculated as Φ_PSII_ = (F_m_’—F_s_)/F_m_’.

### 4.3. Chlorophyll Content Determination

Leaves (0.05 g) were ground with liquid nitrogen in a 10 mL centrifugal tube and extracted in 4 mL of 80% acetone at 4 °C in the dark overnight. With 80% acetone as the blank control, the absorbance of supernatant was measured by a UV-Vis 2450 spectrophotometer (Shimadzu, Kyoto, Japan) at wavelengths of 663 nm and 645 nm. According to Wellburn [36], chlorophyll and carotenoid contents were calculated.

### 4.4. Hydrogen Peroxide (H_2_O_2_) Histochemical Staining and H_2_O_2_ Content Measurements

3,3′-Diaminobenzidine (DAB) staining was used to detect the production of H_2_O_2_ in situ. Fresh leaves were infiltrated with 50 mM potassium phosphate buffer (pH 7.0) containing 0.5 mg mL^−1^ DAB and exposed to a vacuum for 20 min. After incubation for 2 h in the dark, 95% (*v*/*v*) ethanol was used to eliminate the chlorophyll in the leaves.

Fresh leaves (0.1 g) were weighed and homogenized in cold with 1 mL phosphate buffered saline (50 mM, pH7.0). The homogenate was subsequently centrifuged at 4000 rpm for 15 min at 4 °C. The determination H_2_O_2_ content was measured according to the instructions of the kit (Shenzhen Ziker Biological Technology Co., Ltd., Shenzhen, Guangdong, China). The absorbance of the sample was determined at a wavelength of 450 nm with a multimode plate reader (EnSpire, PerkinElmer, Waltham, MA, USA).

### 4.5. Cell Membrane Leakage Rate and Malondialdehyde (MDA) Content Analysis

Cell membrane leakage was measured by a DDS-11C conductometer (Shanghai Dapu Instruments) as described by Zheng et al., with minor modifications [37]. Three 0.8-cm-diameter leaf discs were incubated in 5 mL of distilled water for 3 h. Then, the samples were boiled for 30 min. The cell membrane leakage rate was calculated according to the formula: (R1/R2) × 100%, where R1 and R2 are the initial conductivity and electrolyte conductivity, respectively.

Malondialdehyde (MDA) was determined using the thiobarbituric acid (TBA) method. Leaf samples (0.1 g) were ground in 2 mL of 0.1% (*w*/*v*) trichloroacetic acid (TCA) solution, and the homogenate was centrifuged. Total of 1 mL of supernatant was reacted with an equal volume of 0.67% (*w*/*v*) thiobarbituric acid (TBA) in a boiling water bath for 30 min. The absorbance of the mixture was measured at 600, 532, and 450 nm wavelengths by a UV-Vis 2450 spectrophotometer. According to the formula of Wang and Jin [38], the MDA content was calculated.

### 4.6. Enzymatic and Non-Enzymatic Antioxidants Determination

Flavonoid, total phenol, and total antioxidant capacity (TAC) were extracted from 0.1 g of leaves in 2 mL of 95% (*v*/*v*) methanol at 4 °C for 24 h. Briefly, flavonoid content was measured as described by the modified method of Park et al. [39]. The reaction mixture contained 2 mL of 8-fold diluted extract, 200 μL of 5% NaNO_2_, 300 μL of 10% AlCl_3_, and 1 mL of 1 M NaOH. The absorbance of the mixture at 510 nm was determined by using a spectrophotometer. According to Cheung et al. [40], the total phenolic content of the extracts was measured following the method of Folin–Ciocalteu at a wavelength of 765 nm. 1 mL 10% Folin–Ciocalteu Reagent and 2 mL of 0.7 M Na_2_CO_3_ were mixed with 1 mL of extract. Total antioxidant capacity was assayed in a reaction solution consisting of 150 μL of extracts and 3 mL of 120 mM DPPH solution [41]. The absorbance at 517 nm was determined after the mixture was incubated in the dark for 20 min.

Enzymatic antioxidants including SOD, POD, CAT, and APX were determined according to the methods of Tan et al. [42], Du et al. [43], Jiang et al. [44], and Nakano and Asada [45], respectively, with slight modifications. SOD activity was measured by examining its ability to inhibit the photoreduction of nitro blue tetrazolium (NBT). Each 3 mL assay mixture contained: phosphate buffer (50 mM; pH 7.8), 20 μM riboflavin, 130 mM methionine, 0.1 μM EDTA, 750 mM NBT, and 100 μL of enzyme extract. The absorbance of the samples was recorded at a wavelength of 560 nm. The reaction solution for POD activity comprised phosphate buffer (50 mM, pH 7.0), 30 mM H_2_O_2_, guaiacol, and 100 μL of enzyme extract. The enzyme activity was estimated by recording the increase in absorbance at 470 nm every 15 s for 3 min. The CAT activity was measured by catalyzing the breakdown of H_2_O_2_ at 240 nm during 2 min. A final volume of 3 mL of assay mixture included 30 mM H_2_O_2_ and 100 μL of enzyme extract. For the estimation of APX activity, reaction ingredients were composed of 50 mM phosphate buffer (pH 7.0), 0.1 mM EDTA, 5 mM AsA, and 20 mM H_2_O_2_. Subsequently, the reaction was started after which 100 μL of enzyme extract was added, and the change in absorbance at 290 nm was recorded for 1 min.

### 4.7. Gene Expression Analysis

Total RNA was extracted using TRIzol reagent (Invitrogen, California, MA, USA) following the manufacturer’s protocol. Complementary DNA (cDNA) was synthesized using a PrimeScript RT reagent kit (TaKaRa, Shiga, Japan)). Quantitative reverse transcription polymerase chain reaction (qRT-PCR) analysis was achieved by a Bio-Rad CFX96 Real-Time PCR System (CFX96, Bio-Rad, California, USA) using a SYBR Premix Ex Taq™ II Kit (Takara, Tokyo, Japan). The cycle conditions consisted of initial denaturation at 95 °C for 30 s, followed by 39 cycles at 95 °C for 5 s and 60 °C for 34 s, and a final melt-curve of 65–95 °C. The analysis of gene expression levels was conducted by the 2^−ΔΔ*C*t^ method [46]. The glyceraldehyde-3-phosphate dehydrogenase (*GAPDH*) gene was used as an internal reference gene in this study. The primers for each gene used for qRT-PCR assays are presented in Table 1.

### 4.8. Statistical Analysis

In this experiment, all of the measurements are shown as the means ± standard errors (SEs) of at least three biological replicates. Data were evaluated by one-way analysis of variance (ANOVA) by using IBM SPSS^®^ Statistics (Version 19.0, IBM Corporation, New York, NY, USA). Two-tailed Student’s *t*-test was used to compare the response of two Sphagneticola species under HT treatments. Differences between groups were considered statistically significant if the *p* value was less than 0.05 (*p* < 0.05). OriginPro (Version 8.0, OriginLab Corporation, Northampton, MA, USA) and Adobe Photoshop CC (Version 2014, Adobe Systems, San Jose, CA, USA) software were used for regression analyses and image processing, respectively.

## Figures and Tables

**Table 1:** Primer sequences of internal and target genes.

Gene Name	Primer Sequence
*GAPDH*	Forward: 5′-CTGCTTCATTCAACATC-3′
	Reverse: 5′-CTCACGGTCAGATCAACA-3′
*PsbP*	Forward: 5′-TGCAGCAAGGGATAAGGATGT-3′
	Reverse: 5′-ACAAATGAAAGAGCATGAACAAAGA-3′
*PsbA*	Forward: 5′-TGGAGGAGCAGCAATGA-3′
	Reverse: 5′-GCGAAAGCGAAAGCCTA-3′
*RubiscoL*	Forward: 5′-CGGTCTCTCCAGCGCATAAA-3′
	Reverse: 5′-CGCCTCACGGTATCCAAGTT-3′
*SOD*	Forward: 5′-TGGTTTGAAAGCGGTGG-3′
	Reverse: 5′-CTGGTTTAAGCCCTGTGAT-3′
*POD*	Forward: 5′-CAACACCGCAGAGAAAGACT-3′
	Reverse: 5′-CTGGGAGGTAAAGAGAAC-3′
*CAT*	Forward: 5′-CAAGACCTGGCCTGAG-3′
	Reverse: 5′-TGTCTCTGAGTGTCCG-3′
*APX*	Forward: 5′-CTAAGGAAGGCAGACTC-3′
	Reverse: 5′-CCTGATCTATCTGCATGTG-3′
